# Influence of the crystallographic texture of ITO on the electrodeposition of silver nanoparticles†

**DOI:** 10.1039/d3ra00577a

**Published:** 2023-02-23

**Authors:** Yorick Bleiji, Mees Dieperink, Imme Schuringa, Hongyu Sun, Esther Alarcon-Llado

**Affiliations:** a Center for Nanophotonics Amsterdam The Netherlands e.alarconllado@amolf.nl

## Abstract

The electrochemical control over nucleation and growth of metal nanoparticles on foreign substrates is an active field of research, where the surface properties of the substrate have a key role in nucleation dynamics. Polycrystalline indium tin oxide (ITO) films are highly desired substrates for many optoelectronic applications, for which the only parameter that is often specified is the sheet resistance. As a result, growth on ITO is highly irreproducible. Here, we show that ITO substrates with same technical specifications (*i.e.* sheet resistance, light transmittance and roughness) and supplier may still have different crystalline texture, which we find it has a strong impact on the nucleation and growth of silver nanoparticles during electrodeposition. We find that the preferential presence of lower index surfaces leads to few orders of magnitude lower island density, which is strongly dependent on the nucleation pulse potential. By contrast, the island density on ITO with preferential 〈111〉 orientation is barely affected by the nucleation pulse potential. This work highlights the importance of reporting the surface properties of polycrystalline substrates when presenting nucleation studies and metal nanoparticle electrochemical growth.

## Abstract 2

This work highlights the importance of reporting the surface properties of polycrystalline substrates when presenting nucleation studies and metal nanoparticle electrochemical growth.

## Graphical abstract

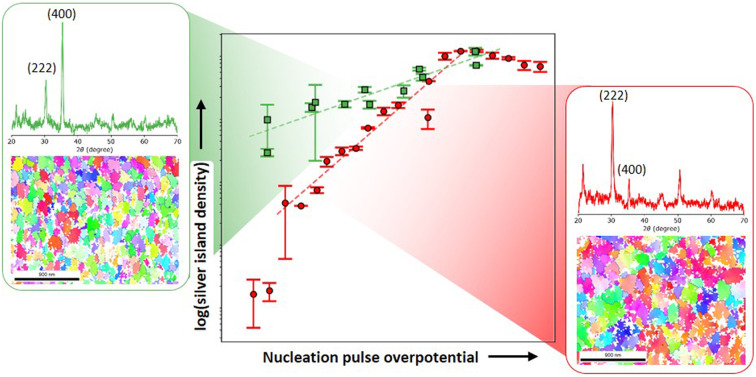

Nanometer-scale metal particles exhibit chemical and physical properties which differ significantly from macroscopic metal phases, including strong interaction with visible light due to their localized surface plasmon resonances. This strong optical response creates bright colors that can be fine-tuned by the nanoparticle size and composition. In particular, silver nanoparticles (NPs) have received extensive attention since it can support a strong surface plasmon polariton modes resulting in high quality factors, which can be tuned all across the visible and NIR regime.^1^ This strong and tuneable interaction of visible light with silver NPs has found applications in sensing,^2^ surface enhanced Raman spectroscopy (SERS),^3,4^ smart windows,^5–8^ dynamic color displays,^9^ plasmonic color filters,^10^ photo(electro)catalysis^11,12^ and more.^13^ For many of these applications, it is essential to reliably and precisely control the size and density of silver NPs on conducting transparent supports, such as indium tin oxide (ITO).

Electrochemical deposition is an attractive growth method owing to its inherent low cost, minimal raw material usage, low temperatures and high throughput. Electrochemical deposition of silver on ITO naturally leads to scattered, low density irregular island growth owing to the low surface energy and high nucleation barrier of ITO, which poses a challenge for smooth continuous deposits. Several works have shown that cyanide compounds and other organic additives in the electrolyte solution can lead to high density island formation, which can be controlled by a nucleation pulse step.^14–17^ However, the high toxicity and environmental impact of such electrolytes demands the search for more sustainable alternatives, such as thiosulfate baths,^18^ non-toxic organic additives,^19^ or even plant extracts.^20^ While the nucleation kinetics and growth dynamics of silver on ITO are well studied for cyanide-based electrolytes,^14,15^ nucleation studies with non-toxic and sustainable electrolytes are scarce.^4,5,9,21,22^

In all these works, attention is given to the nucleation pulse parameters (*i.e.*, pulse potential and time) on island density and size, while the influence of the surface properties of the ITO substrate are highly omitted. The uncertainty in the physico-chemical properties of commercial ITO (*e.g.* chemical composition, roughness or crystalline orientation) is typically reduced down to reporting the sheet resistance and average optical transmission. There is no straightforward connection between these characteristics and the ITO surface properties, despite it is well known that the substrate surface plays a key role in the nucleation and growth dynamics.

In this work we elucidate the impact of crystallographic texture of ITO substrates with the same nominal specifications (*i.e.* roughness, sheet resistance and supplier) on silver nucleation dynamics in a silver sulphate based aqueous solution. We find that the nucleation density as a function of nucleation pulse potential can differ by several orders of magnitude depending on the surface properties (*i.e.* the preferred ITO crystal orientation, grain size, and grain misorientation angle). In particular we find that the ITO from batch 2 having small randomly oriented grains, leads to a high nuclei density and diffusion coupled growth that is barely affected by the nucleation pulse potential. By contrast, ITO from batch 1 having larger grains with strong preference for low Miller index surfaces, leads to island densities that can be tuned by the nucleation pulse potential. This work highlights the importance of reporting the surface properties of ITO substrates for improved reproducibility of metal NPs growth.

## 1 Experimental

### ITO substrates

Two sheets of ITO coated glass (KinTec, nominal sheet resistance 10–15 Ω sq.^−1^, 150 × 150 mm^2^) were cut into 25 × 25 mm^2^ pieces and used as working electrodes (exposed area 0.95 cm^2^). Pieces taken from the first sheet of ITO shall be referred to as batch 1, and similarly ITO pieces taken from the second sheet are referred to as batch 2. The average root mean square (rms) surface roughness of two batches are 2.0 ± 0.1 and 3.4 ± 0.2 nm, respectively, as measured with AFM. Despite the relatively large range for the specified nominal sheet resistance, we measured *R*_sh_ = 15.8 ± 0.2 Ω sq.^−1^ and *R*_sh_ = 14.8 ± 0.5 Ω sq.^−1^ for batch 1 and 2, respectively.

**Fig. 1:**
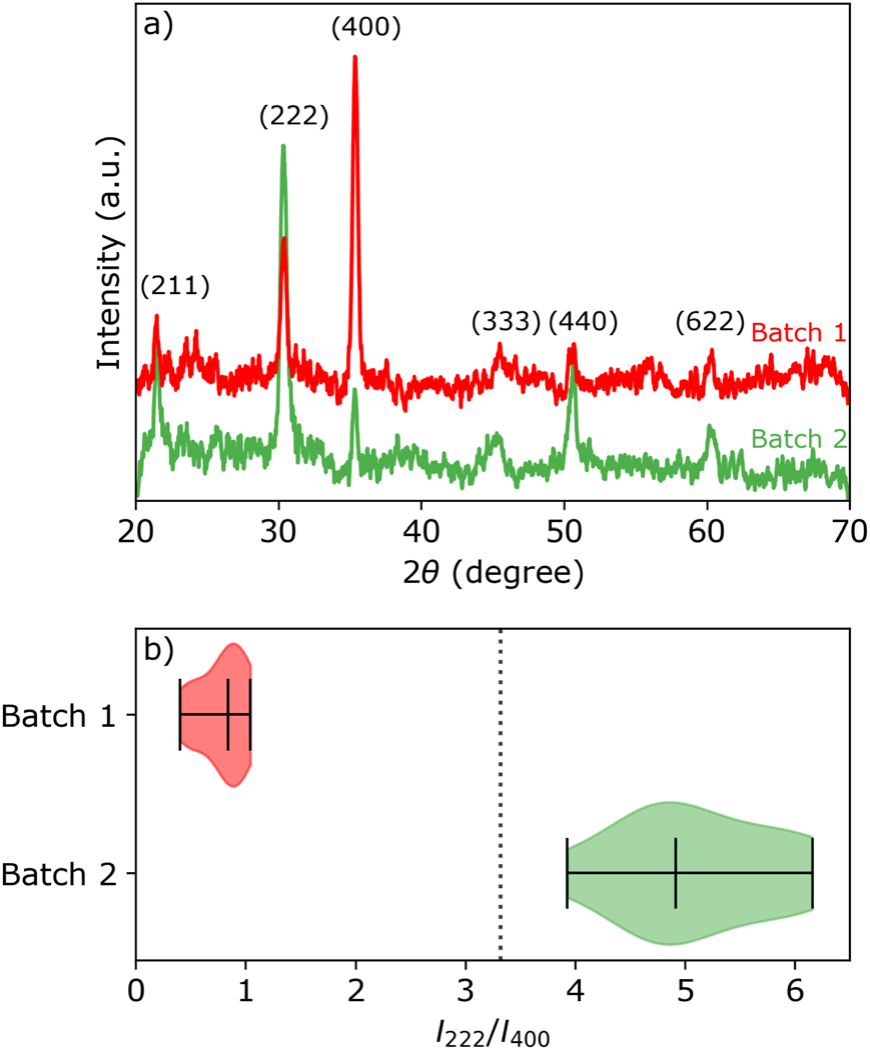
(a) 2*θ* scans of representative batch 1 (red) and batch 2 (green) samples with the corresponding crystallographic orientations. (b) Violin plot of the peak intensity ratio distribution between the (222) and (400) peaks for samples from each batch. The distribution medians and limits are marked by the bars. The vertical dotted line represents the value of the theoretical ratio *I*_222_/*I*_400_ for powder diffraction of In_2_O_3_.

XRD and electron back-scattered diffraction (EBSD) analysis (Fig. 1a and Section S1 in the ESI†) reveal that the ITO pieces from batch 1 have a strong preference for the (400) orientation, while the ITO pieces from batch 2 have near random oriented grains with a slight preference for the (222) orientation. All ITO pieces within the same batch show similar XRD patterns, represented by the intensity ratio *I*_222_/*I*_400_ of the (222) and (400) peaks in Fig. 1b. The theoretical peak intensity ratio *I*_222_/*I*_400_ is represented by a vertical black dashed line. The theoretical peak intensity ratio was simulated for the In_2_O_3_ crystal structure (bixbyite, space group 206, *Ia*3̄, *a* = 10.117 nm) using CaRIne Crystallography version 3.1.

### Substrate preparation

The ITO substrates were cleaned by brushing with soap and ethanol to remove any residual glue that was used for cutting the samples. After, the ITO substrates were sonicated for 10 min in ultra pure water, 10 min in acetone and 5 min in isopropanol (IPA). The ITO substrates were treated with ozone for 15 min (UV/ozone ProCleaner BioForce Nanosciences) to remove any organics and to activate the surface just before the start of the experiment,^23^ resulting in a more hydrophilic surface.

### Electrochemistry

A custom build Teflon cell of 24 mL volume was used, using a standard three-electrode configuration. A Pt disc (exposed area 3.08 cm^2^) was used as counter electrode, and an Ag/AgCl electrode (leakless miniature ET072, EDAQ) was used as reference electrode. The miniature reference electrode was calibrated against a saturated Ag/AgCl reference electrode (XR300, Hach) just before the start of each experiment. All experiment were performed using a SP-300 Bio-Logic potentiostat.

An aqueous (Millipore Milli-Q®, *ρ* > 18.2 MΩ cm) electrolyte containing 1 mM Ag_2_SO_4_ (99.999%, Sigma-Aldrich), 1 mM saccharin (≥99%, Sigma-Aldrich), 100 mM Na_2_SO_4_ (≥99.0%, anhydrous, granular, Sigma-Aldrich), 38 mM H_2_SO_4_ (96% solution in water, ACROS Organics) (pH of 1.7) was used for the electrodeposition of silver. The solution resistance *R*_s_ was determined by electrochemical impedance spectroscopy and was found to be 25 ± 5 Ω. The solution resistance value was used for the *IR* correction of the applied potential. A double pulse method was used to perform the silver deposition.^15,19^ A nucleation pulse *E*_n_ was applied for 30 ms followed by a growth pulse *E*_g_ = +0.373 V *vs.* Ag/AgCl for 20 s, which was taken from Kung *et al.*^19^ The nucleation potential *E*_n_ was varied between +0.2 and −0.883 V *vs.* Ag/AgCl. Fig. S5† shows a schematic of this potential scheme, where the lowest and highest potentials are indicated by a dashed green and solid red line, respectively. All experiments were performed at room temperature (18–21 °C) without stirring. For each experiment a fresh ITO sample was used which was cleaned as described above. Directly after deposition, the samples were rinsed in ultra pure water and IPA to remove any precipitation and were dried using a flow of nitrogen gas.

### Characterisation

Morphological and structural characterization of the deposited silver nanoparticles was performed using a FEI Verios 460 scanning electron microscope (SEM), operated at 5 kV and 100 pA, using a working distance of 4 mm. Topographical maps were obtained with atomic force microscopy (AFM), using a Bruker Dimension Icon and a ScanAsyst-Air probe (Bruker, nominal tip radius 2 nm). Details on SEM image processing can be found in Section S4 of the ESI.†

X-ray diffraction (XRD) was performed on most ITO substrates using a Bruker D2 Phase diffractometer. The Cu kα irradiation was operated at 30 kV and 10 mA. The substrates were scanned between 2*θ* = 25° and 70°, with 0.016° increment using a dwell time of 0.1 s.

Electron back scatter diffraction (EBSD) patterns were collected in a FEI Verios 460 SEM, operated at 10 kV and 800 pA using a working distance of 8.2 mm, using an Amsterdam Scientific Instruments Timepix detector. The EBSD maps were obtained using a sample tilt of 70° relative to the electron beam, an exposure time of 10 ms, and a step size of 20 nm. The collection and analysis is done using the software OIM Analysis of EDAX.

## 2 Results and discussion

**Fig. 2:**
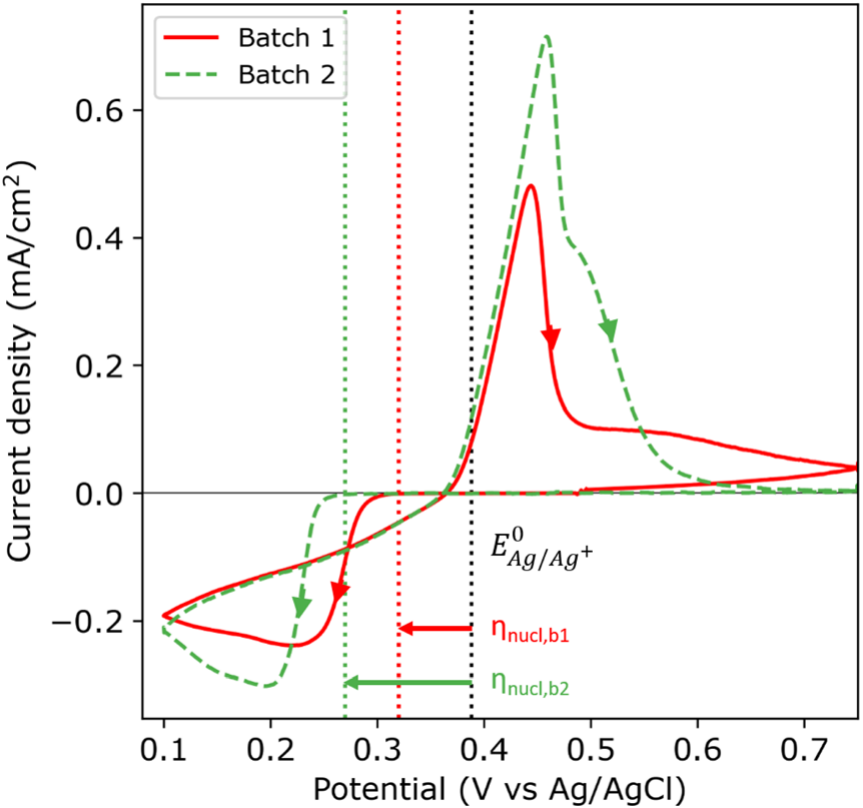
First cycle of the cyclic voltammogram (25 mV s^−1^) for the silver sulphate electrolyte containing saccharin on ITO batch 1 (red solid) and batch 2 (green dashed). The arrows indicate the scan direction. The nucleation onset potential is indicated with red and green dotted lines for batch 1 and 2, respectively. The black dotted line indicates the experimentally found Nernst equilibrium potential 
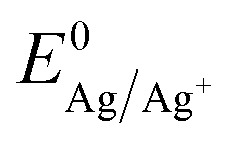
.

Fig. 2 shows the first cycle of the cyclic voltammogram (CV) for silver deposition in aqueous electrolyte on two nominally equal sheets of ITO (see Experimental section). The CV is initiated at open circuit potential (OCP, 0.49 and 0.47 V *vs.* Ag/AgCl for batch 1 and 2, respectively) and scanned with a scan rate of 25 mV s^−1^ towards the cathodic direction, as indicated by the arrows. During the first cycle, silver is deposited onto the clean ITO surface upon overcoming the onset potential for silver nucleation, as indicated by the sharp increase in cathodic current (marked by the red and green dashed vertical lines). At more negative potentials, the cathodic current rapidly increases after nucleation and reaches a maximum, characteristic for diffusion limited growth.^24^

The onset potential for silver nucleation (0.32 and 0.27 V *vs.* Ag/AgCl for batch 1 and batch 2, respectively) is found to be more negative than the experimentally determined Nernst equilibrium potential of silver 
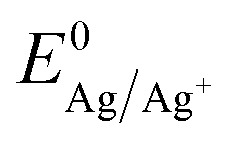
 (0.39 V *vs.* Ag/AgCl). The difference between the onset potential and the Nernst equilibrium potential is known as the nucleation overpotential *η*_nucl_,^25^ which is typical in deposition onto foreign non-wetting substrates. The fact that the nucleation potential is the lowest for batch 1 is in agreement with its preferential crystallographic orientation, since the ITO from batch 1 has more higher surface energy planes in comparison to the ITO from batch 2 (*γ*(400) > *γ*(222)).^26,27^ Nucleation on higher energy surfaces is expected to be thermodynamically more favourable, and hence occurs at reduced nucleation overpotentials. The ratio of the (222) to (400) peak intensity in batch 2 (see Fig. 1) indicates that these substrates have significantly less (400)-oriented surface area compared to batch 1. One might thus expect that the reductive current due to nucleation on the (400)-oriented surface in batch 2 is negligible and most of the current is due to nucleation on (222)-oriented surface, which takes place at a larger overpotential.

During the reverse scan in the cyclic voltammogram, the current transient crosses the forward scan (*i.e.* crossover) in both cases. This is an indication of nucleation and growth, where growing nuclei result into a larger surface area and hence an increase in current, and deposition onto the already formed nuclei does not require an overpotential.^28^ The total amount of transferred charge and hence the amount of deposited silver is virtually the same for both batches (2.61 and 2.59 mC cm^−2^ for batch 1 and 2, respectively). In both batches, the current flips from cathodic to anodic at 0.37 V *vs.* Ag/AgCl, and defines the onset for silver dissolution. Integrating over the anodic peak current, we find that more silver is being stripped from batch 2 compared to batch 1 (2.07 and 2.54 mC cm^−2^ for batch 1 and 2, respectively). During the second cycle of the CV (see Fig. S6 in the ESI†), the nucleation overpotential of silver deposition on batch 1 is near zero due to the incomplete stripping (79%). Silver nuclei are still present on the surface of batch 1 at the start of the second cycle, and therefore the nucleation and growth can occur at near zero nucleation overpotential. However, the stripping of silver was more complete in batch 2 (98%). Therefore, almost no silver nuclei are present on the surface at the end of the first cycle, hence the large nucleation overpotential in the second cycle.

In the following, we study the effect of the nucleation pulse potential in the double-pulse method^15,19^ on the nucleation and growth of silver NPs on the two types of ITO substrates. In order to qualitatively compare the nucleation kinetics in both types of ITO, we convert all applied voltages to the overpotential relative to the nucleation overpotential *η*_nucl_ of the respective batch. As such, the nucleation pulse overpotential *η*_n_ is defined as:

1 *η*_n_ = |*η*_n,*IR*_ − *η*_nucl_|

where2
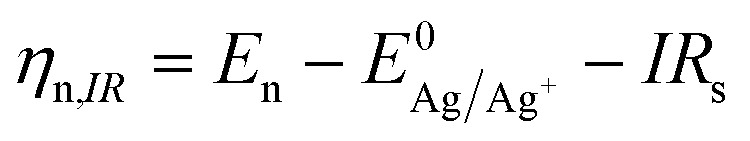
where *E*_n_ is the applied nucleation pulse potential, 
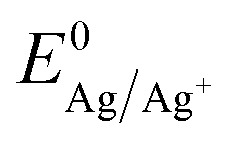
 is the Nernst equilibrium potential, *I* is the current during the nucleation pulse, and *R*_s_ is the solution resistance. Since the nucleation overpotential *η*_nucl_ is defined as the difference between the onset potential and the Nernst equilibrium potential, eqn (1) can be simplified to:

3 *η*_n,*IR*_ = |*E*_n_ − *IR*_s_ − *E*_nucl_|

Note that the exact value of the Nernst equilibrium potential is therefore not relevant for the value of the nucleation pulse overpotential, since the term drops out from the equation.

**Fig. 3:**
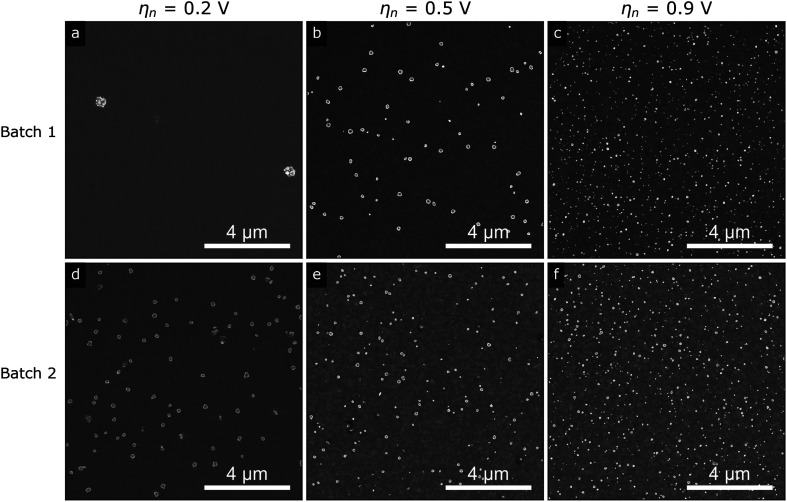
Representative SEM images of the silver islands on ITO from (a)–(c) batch 1 and (d)–(f) batch 2 using a nucleation pulse overpotential of (a) and (d) 0.2 V, (b) and (e) 0.5 V and (c) and (f) 0.9 V.

Fig. 3 shows representative SEM images of the silver islands on ITO obtained at nucleation pulse overpotentials of 0.2 V (a) and (d), 0.5 V (b) and (e) and 0.9 V (c) and (f), for the two different batches of ITO substrates (top and bottom rows). In line with electrochemical theory for kinetically controlled nucleation,^14,29,30^ we observe that the island density increases with increasing nucleation pulse overpotential regardless of substrate, owing to the increased electrodeposition kinetics as the potential is increased. At the same time, we observe that the size of the islands decreases with increasing density (see Fig. S8†), likely as a result of higher ion competition between nuclei as the number of nuclei increases. In fact, since we kept the growth pulse constant for all samples, the mean island radius is fully determined by the island density (see Fig. S9 in the ESI†).

Despite the qualitative agreement between the trends for both substrates and the fact that electrochemical parameters were carefully kept constant throughout the study, the island density is quantitatively not reproduced for the two ITO batches with same nominal specifications (roughness, sheet resistance and supplier, see Experimental section and ESI†). This is most evident by comparing the images in Fig. 3a and d, both deposited at a low nucleation pulse overpotential. Such inconsistencies between growths on presumably equivalent substrates makes reproducibility of published works difficult and may hamper the overall understanding of silver (and any other metal) nucleation on ITO.

**Fig. 4:**
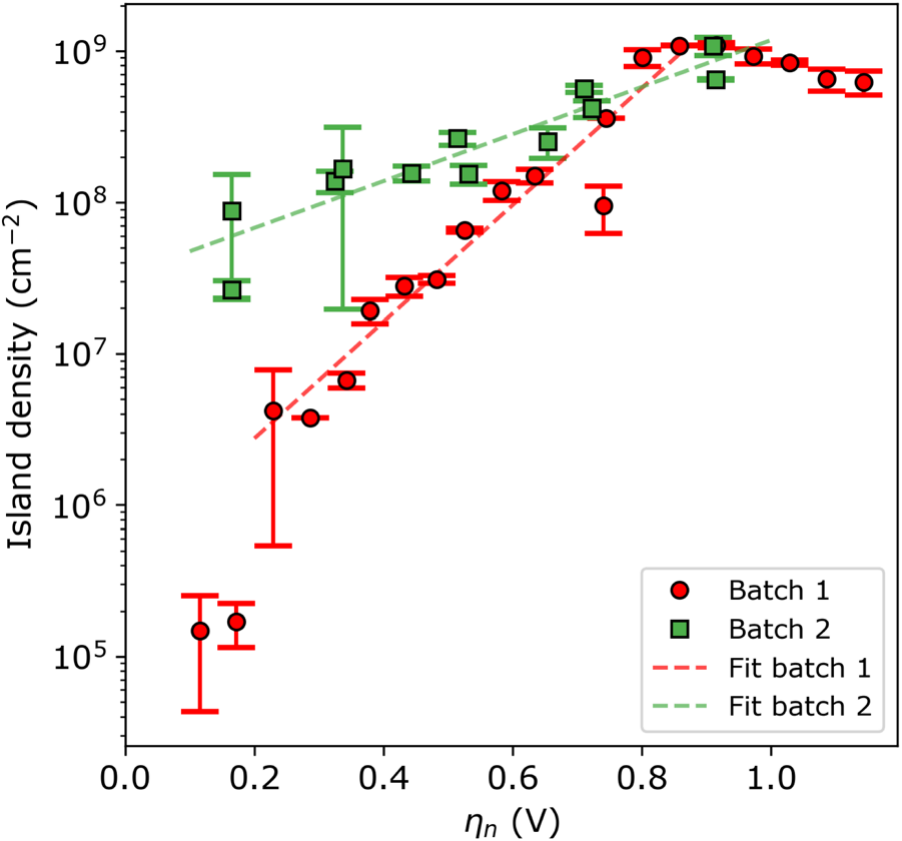
Island density *versus* nucleation pulse overpotential for batch 1 (red circles) and batch 2 (green squares). The red and green dashed lines are the fits to the data points of batch 1 and 2, respectively. The inverse slope *B* is 250 ± 23 and 625 ± 84 mV dec^−1^, and the pre-exponential factor *A* is 4.7 × 10^5^ ± 2.2 × 10^5^ and 3.4 × 10^7^ ± 9.5 × 10^6^ cm^−2^ for batch 1 and 2, respectively. Note that a logarithmic scale is used for the vertical axis.

For a more quantitative assessment, Fig. 4 shows the island density as a function of nucleation pulse overpotential, as extracted from the SEM images. The samples corresponding to batch 1 and 2 are shown in red circles and green squares, respectively. From the semi-logarithmic representation, one can see that for *η*_n_ < 0.8 V the island density exponentially increases with increasing nucleation pulse overpotential for both substrates. However, the rate of increase is much more pronounced for batch 1 compared to batch 2, which leads to a difference of 2 to 3 orders of magnitude between particle densities for the same (low) nucleation pulse overpotential.

Previous studies have empirically shown^30–35^ that an exponential relationship exists between the island density and overpotential following:4
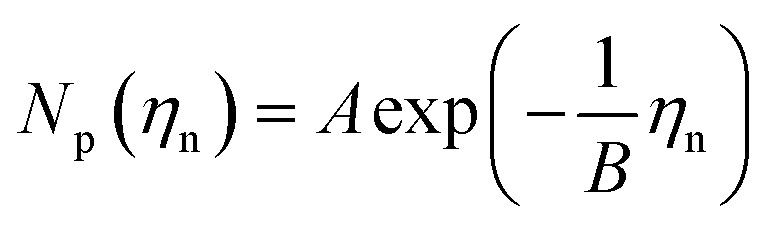
Here, *A* and *B* are voltage independent empirical parameters, where the pre-exponential factor *A* is thought to be related to the surface density of available sites at OCP, and the exponential component *B* is an activation term related to the electron kinetics. Eqn (4) is valid for the assumption of instantaneous nucleation or progressive nucleation where the nucleation pulse duration is sufficient long enough to nucleate on all active sites. Fitting our data to the equation above, we find the inverse slope *B* is 259 ± 20 and 645 ± 84 mV dec^−1^, and the pre-exponential factor *A* is 4.7 × 10^5^ ± 1.8 × 10^5^ and 3.3 × 10^7^ ± 9.0 × 10^6^ cm^−2^ for batch 1 and 2, respectively. Important to note that the two data points at the lowest nucleation pulse overpotentials were left out of the fitting procedure, since the duration of nucleation pulse was not long enough to complete nucleation at such low nucleation pulse overpotentials. In other words, the island density was still not saturated within the duration of the nucleation pulse (incomplete progressive nucleation). We therefore find that batch 1 has a greater tunability by nucleation pulse overpotential than batch 2.

At nucleation pulse overpotentials larger than 0.8 V, the island density saturates in the order of 10^9^ cm^−2^, in both types of substrates. Considering the classical nucleation and growth models, a saturated island density with increasing nucleation pulse overpotential indicates that nucleation is limited by mass transport, a condition that depends on the properties of the electrolyte and the electrochemical cell.^28,30,36,37^ Under such circumstances, the island density saturates once the diffusion zones of the islands overlap, preventing the formation of new nuclei and resulting in a constant mean island radius with increasing nucleation pulse potential (see Fig. S10†). Next to the limitations by mass transport, spurious hydrogen evolution at high cathodic potentials may poison the ITO surface hereby reducing the amount of active sites for silver nucleation. This effect explains the small decrease in island density at large nucleation pulse overpotentials.

We verify the mass transport limited island density through a spatial correlation analysis using the nearest-neighbour distance (nnd). In the case where nucleation and growth of the islands is not limited by mass transport, the nnd distribution obeys complete spatial randomness (CSR).^36^ In other words, in a random point pattern the probability of finding *n* points (*i.e.* islands) within a certain distance from an arbitrary island is Poisson distributed, and has a well-defined probability density function (pdf) *p*(*r*):^36,38^

5 *p*(*r*) = 2π*ρr* exp(−π*ρr*^2^)

where *r* is the distance from the central point, and *ρ* is the number of points per unit area (*i.e.* island density).

**Fig. 5:**
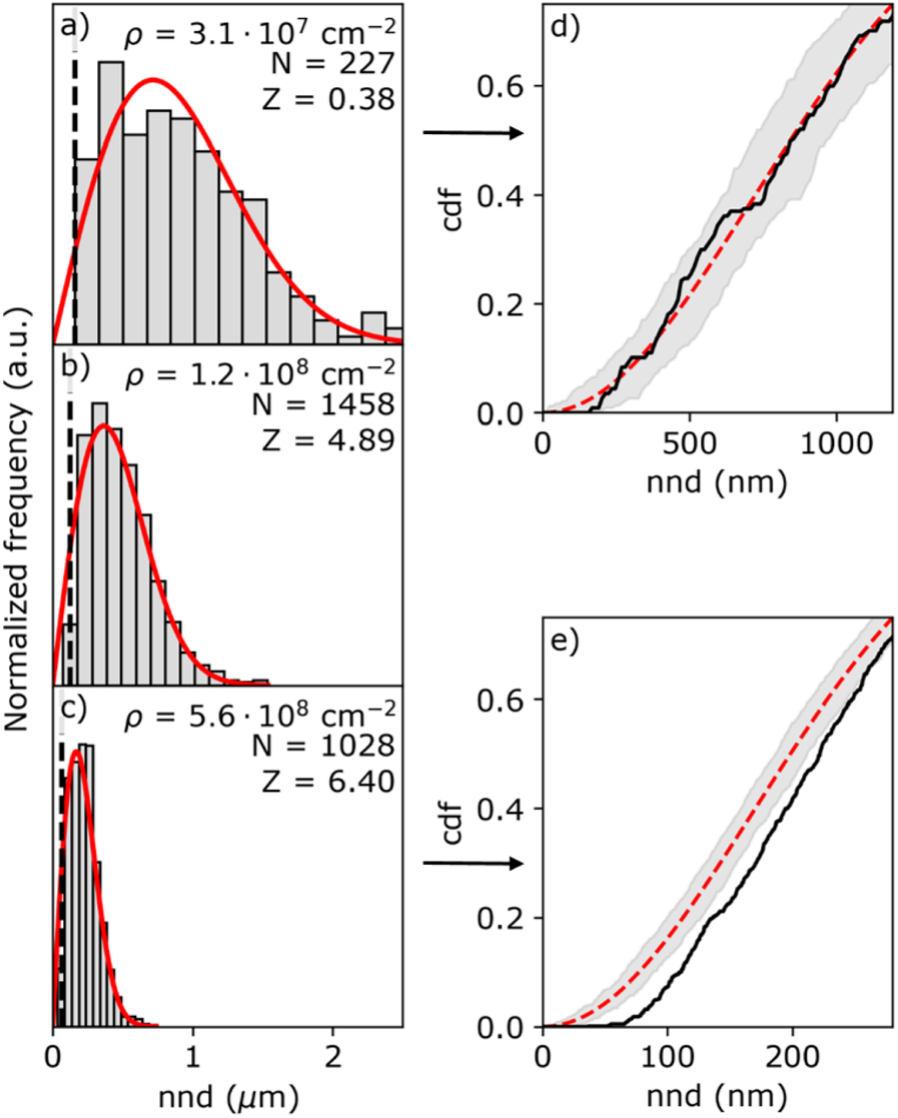
Histograms of the (a)–(c) nnd obtained from SEM images for the island densities of (a) 3.1 × 10^7^ cm^−2^ (batch 1, *η*_n_ = 0.46 V), (b) 1.2 × 10^8^ cm^−2^ (batch 1, *η*_n_ = 0.55 V), and (c) 5.6 × 10^8^ cm^−2^ (batch 2, *η*_n_ = 0.67 V). The histograms are normalized and compared to the Poisson distribution (red solid line). The black dashed line in (a)–(c) indicates the mean diameter of the islands. The island density *ρ*, number of islands *N*, and the *Z* value are listed in each histogram. (d) and (e) Are the cumulative distribution functions of (a) and (c), respectively. The shaded area indicates the 95% confidence interval.

Fig. 5 shows three representative nnd distributions for three different island densities from low (a) to high (c). The solid red line indicates the Poisson distribution as given by eqn (5). Due to the finite size of the islands, the histogram is distorted at short nnd, as indicated by the black line representing the mean common island diameter. To verify the results from the first nnd distribution analysis, we also analyzed the second nnd (*i.e.* next nearest neighbours) distribution where these short nnd are less important due to the *r*^3^ dependency of the pdf function (see Section S7 in the ESI†). From the histograms, it seems that the nnd obeys CSR in all cases. However, the high island density distributions lack close neighbouring particles. This effect is more visible in the cumulative distribution function (cdf) of the highest and lowest particle density, shown in Fig. 5d and e. While the cdf of the lowest density nnd distribution (black curve in Fig. 5d) falls within the confidence interval of the Poisson cdf (shaded grey area), the cdf of the highest density nnd distribution (black curve in Fig. 5e) clearly falls out of the Poisson confidence interval.

To confirm whether the island patterns obey CSR or not, we perform a *Z*-test, where the *Z*-value is defined as:6
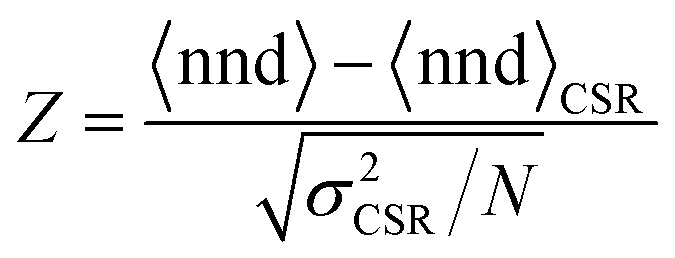
where 〈nnd〉 is the mean value of the nnd distribution, *N* is the number of points in the distribution, and 〈nnd〉_CSR_ and *σ*^2^_*CSR*_ are the mean nnd value and variance of the CSR probability density function. Distributions having a *Z*-value within ±1.96 are considered to be CSR with 95% confidence (two tailed test with *p* > 0.05). Contrarily, if *Z* < −1.96 the distribution is considered to be clustered, while if *Z* > 1.96 the distribution is considered to have a globally ordered structure.^36^ A more detailed derivation of the first and second order of the pdf, and the definition of the mean and variance of the distribution can be found in Section S7 of the ESI.†

**Fig. 6:**
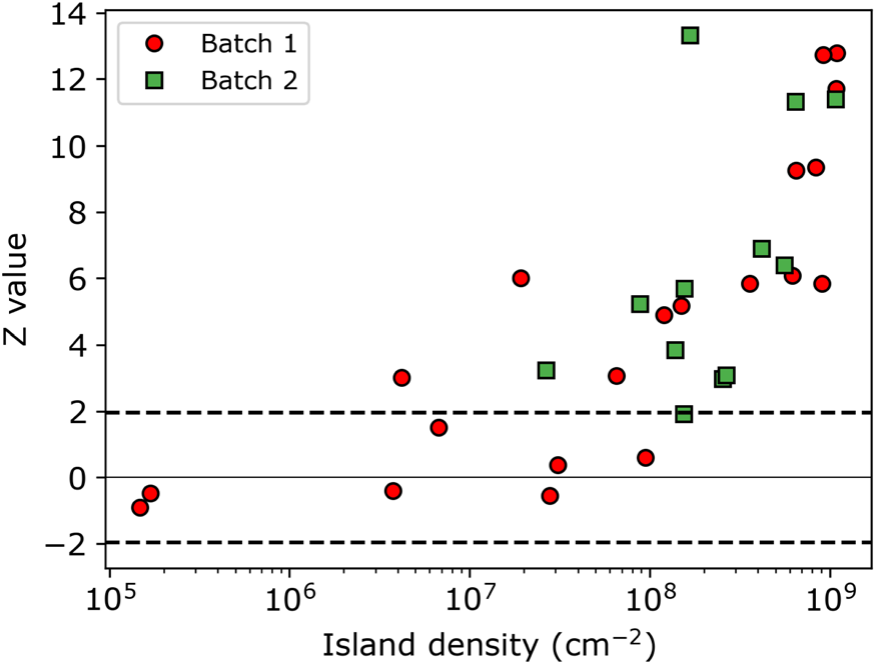
*Z*-Value obtained from individual nnd distributions as function of the island density. Samples from batch 1 and 2 are indicated by red circles and green squares, respectively. The black horizontal dashed lines indicates the region for which the distribution of islands exhibit CSR.

The resulting *Z*-values for the examples shown in Fig. 5a–c are 0.38, 4.89 and 6.4, respectively, which undoubtedly shows that only the spatial distribution of islands having the lowest density is completely random. The *Z*-test analysis of both first and second nnd is performed for all samples, where the first-nnd *Z*-values are shown in Fig. 6 as function of the island density for the two types of substrates (red circles for batch 1 and green squares for batch 2). Second-nnd *Z*-values are shown in Fig. S12.† The *Z*-values for both the first and second nnd distributions indicates CSR at low island densities and up to approximately 10^8^ cm^−2^. For higher island densities the *Z*-value rapidly increases, meaning that the island distribution is becoming globally more ordered. Such an ordered structure is caused by the transition from 3D uncoupled to a 1D diffusion-coupled deposition due to the overlap of diffusion zones around the growing islands.^29,34,38,39^ In the regime of coupled growth, no new islands can nucleate within the diffusion zone and hence forcing an exclusion zone between the islands and leading to a globally more ordered pattern. It is important to note here that almost all of the batch 2 samples fall in the diffusion coupled regime which is in contrast to the batch 1 samples. This partly explains why the inverse slope of the island density *versus* nucleation pulse overpotential is much different to any other values found in literature. Silver nucleation on batch 2 ITO is under a mixed kinetic/diffusion control, leading to an unreasonably large apparent inverse slope *B* (*i.e.* an apparent small electrode kinetics).

Interestingly, a similar conclusion can be drawn from the total transferred charge during growth as function of the island density (see Fig. S11 in the ESI†). Again, the two regimes can be discerned. At low island densities (*i.e.* below 10^8^ cm^−2^), the amount of transferred charge increases with island density. This behaviour agrees well with the uncoupled growth regime. By contrast, at high island densities, the amount of transferred charge saturates. This suggests that at these densities the silver deposition is limited by mass transport instead of by electrode kinetics, which agrees well with the diffusion-coupled growth. In summary, both the nnd and the transferred charge analysis confirm that island density saturation at large nucleation pulse overpotentials arises from mass-transport limitations rather than the surface properties of the ITO substrate, therefore invalidating eqn (4) for assessing nucleation kinetics in batch 2. In fact, we suspect that batch 2 samples have high electrode kinetics, where very small nucleation pulse overpotentials (<100 mV) can lead to very high particle densities.

We have shown that two batches of ITO sheets, even though from the same supplier with same nominal specifications, display a large discrepancy in silver island density for nucleation pulse overpotentials smaller than 0.8 V. Batch 1 samples, which show a preference for (400) crystal orientation having large grain sizes, display the lowest nucleation overpotential and a kinetically-controlled island density that increases exponentially with nucleation pulse overpotential. Contrarily, batch 2 samples with a slight (222) preferential crystal orientation and having smaller grains, show a high nucleation overpotential. However, the fast electrode kinetics lead to such high nucleation densities that nucleation is diffusion limited for almost all nucleation pulse overpotentials as small as 100 mV. As previously mentioned, the lower surface energy in high index surfaces imposes a thermodynamic barrier for growth. Yet, this thermodynamic barrier may be compensated by a high electrode kinetics. In fact, it has been reported that {222}-terminated surfaces of ITO typically show metal sites with incomplete coordination, and are therefore expected to be highly reactive.^40^ As a result, a very high number of nuclei can be formed once the thermodynamic barrier is overcome even with nucleation pulse overpotentials of few tenths of mV. This concept could be extended to similar systems in which the electrochemical reaction is limited by electron kinetics. We therefore expect that the texture of the ITO substrates shall also influence the growth of different materials. Despite the thermodynamic preference for nucleating on the high surface energy facets and on the grain boundaries, from SEM and AFM images we were not able to unequivocally identify if the islands are located on specific facets and/or grain boundaries.

## 3 Conclusion

In this paper, we elucidate the influence of ITO surface orientation on silver NPs growth through a careful and quantitative study on nanoparticle nucleation and growth on two batches of ITO substrates. We use seemingly equal ITO substrates (in terms of sheet resistance, surface roughness and optical transparency) that have a clear different surface texture. We find that in the kinetic regime, silver nucleation dynamics are slower for ITO films with preferential (400) orientation, leading to several orders of magnitude lower silver island densities compared to those found in substrates having a slight preference for the (222) orientation. We elucidate that the empirical exponential relationship between island density and nucleation pulse overpotential is not enough to draw conclusions on electrode kinetics, where additional validation of the growth process is essential. We argue that the enhanced electron transfer rates at {222}-terminated surfaces during the nucleation and growth pulses may be responsible for the larger island density.

Beyond careful control of electrochemical parameters, here we show that neglecting the macroscopic surface characteristics of ITO (and any other polycrystalline substrate) can lead to major ambiguity in nucleation and growth dynamics of metal nanoparticles. Controlling metal particle density and size is crucial in many applications. We show here that XRD characterization prior to deposition is a simple additional measure to tailor metal nanoparticle electrochemical growth on ITO.

## Author contributions

Y. B. and M. D. synthesised the silver particles, I. S. and H. S. performed the EBSD measurements, I. S. performed the EBSD analysis and Y. B. performed all the data analysis. Y. B. and E. A. L. wrote the manuscript. E. A. L. supervised the project.

## Conflicts of interest

There are no conflicts to declare.

## Supplementary Material

RA-013-D3RA00577A-s001
