# Supplementary material for: Influence of the crystallographic texture of ITO on the electrodeposition of silver nanoparticles
Source: RSC Adv. 2023 Feb 23;13(10):6490–7. doi: 10.1039/d3ra00577a (PMC9949353; doi:10.1039/d3ra00577a)
Supplement: RA-013-D3RA00577A-s001 [file RA-013-D3RA00577A-s001.pdf]

Electronic Supplementary Material (ESI) for RSC Advances.
This journal is © The Royal Society of Chemistry 2023

Supplementary Information: Influence of the Crystallographic Texture of ITO on the Electrodeposition of Silver Nanoparticles

S1 EBSD characterisation

Electron back scatter diffraction (EBSD) is a SEM-based technique in which the diffraction patterns (Kikuchi patterns) of the back-scattered electrons are detected. The diffraction pattern is a result of the interaction of inelastically scattered electrons with the crystal structure of the sample, and hence provide information about the type of crystal structure, lattice constant and orientation. EBSD maps were obtained by transforming the Kikuchi lines into points (Hough space), which are fitted using the simplified crystal structure of In_2O_3 (a face-centered cubic $2x2x2$ supercell, space group 225, Fm$\bar{3}$m) using a lattice constant of $a = \frac{1}{2} \cdot 10.12$ Å $= 5.05$ Å.[26] When using the actual crystal structure of In_2O_3 (bixbyite, space group 206, Ia$\bar{3}$) in the fitting procedure, it resulted into pseudo-symmetrical solutions which could not be distinguished. Based on this data the software cannot distinguish the (101) and (110) directions in the crystal, because their difference is too subtle. However, the distinction between the (200) and (444) direction can be made, and their ratio is not affected by fitting the data with the simplified crystal structure.

Figure S1 shows the crystallographic orientation along the z-axis in inverse pole figure (IPF) maps for batch 1 and batch 2. Confidence interval (CI) standardization clean up was used with a CI of 0.1. After two rounds of refitting only the data with CI > 0.1 was kept and used for the determination of grains and grain boundaries. Grains were determined based upon 5° tolerance with a minimal size of 2 pixels. The colors in the map represent the crystallographic orientation perpendicular to the surface.

To get a better insight into the distribution of crystallographic orientations, we represent every pixel from the EBSD map in figure S1 by a dot in the IPF, as shown in figure S2. In line with the XRD spectra shown in figure 1, we find that the surface of batch 1 has a strong preference for the <100> orientation, while the distribution of orientations for batch 2 seems to be more random.

Next to the orientation of the grains, we can also extract the size of the grains from the EBSD maps. We observe a clear difference in the size of the grains between ITO from batch 1 and 2. The average grain size is found to be 0.022 μm^2, and 0.072 μm^2 for batch 1 and 2, respectively. Consequently, the total length of the grain boundaries is larger for batch 2. It is interesting to consider the misorientation of the grains since in general a higher misorientation angle leads to higher grain boundary energies.[41] However, this relation only holds up to a certain misorientation angle, since for larger misorientation angles coincidence take place which results into a maximum of grain boundary energy. Regarding electrochemical nucleation, it would be thermodynamically more favorable to nucleate on the grain boundaries with a higher misorientation angle. We therefore look to the grain boundary length density for small (5-10°), medium (10-20°) and large ($>$ 20°) misorientation angles, which is shown in figure S3. Next to the larger grain boundary length density, batch 2 also has a larger misorientation between the grains. These observations are in line with the observed fast kinetics of batch 2.

To correlate the morphology of the surface with the EBSD maps, we show in figure S4 the overlap between the EBSD map of figure S1a with the corresponding SEM image. It is clear that the morphology of the ITO already reveals the location of the grains. Furthermore, it was found that grains in the <100> and <111> direction could be recognised by the subgrains which are vertically, or horizontally oriented rods, respectively. The morphology of the SEM image can therefore already provide some information about the grain size.

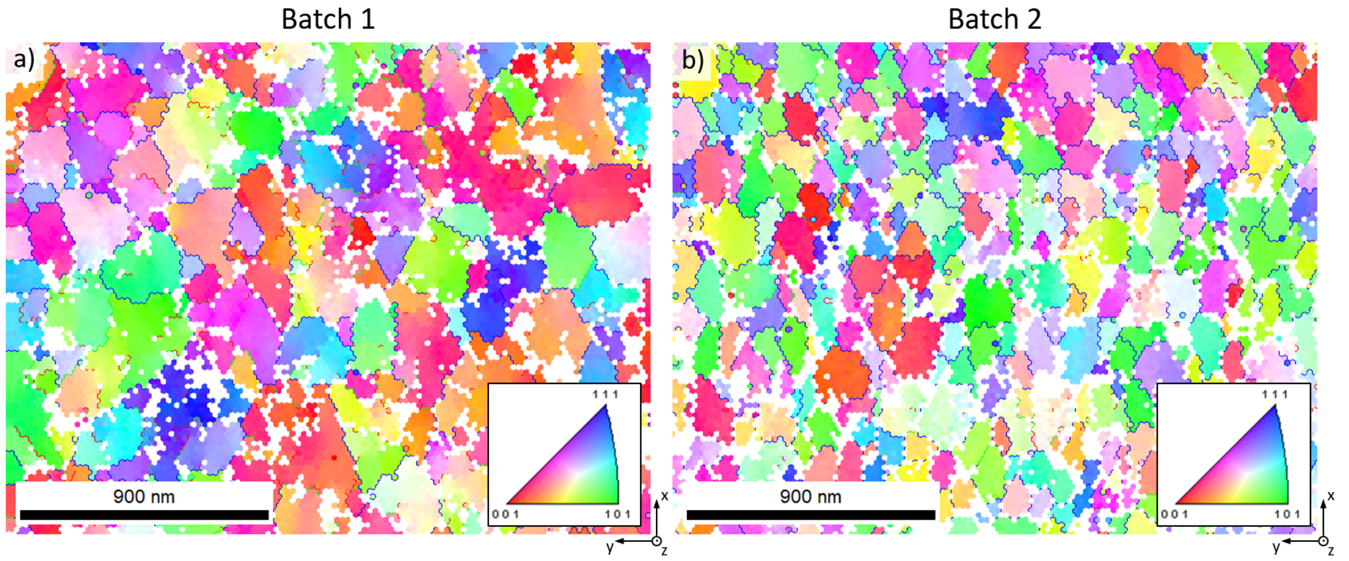

Fig. S1 IPF EBSD maps of a) ITO batch 1 and b) ITO batch 2, showing different crystallographic orientation perpendicular to the ITO surface. The area of both scans is 4.17 μm^2.

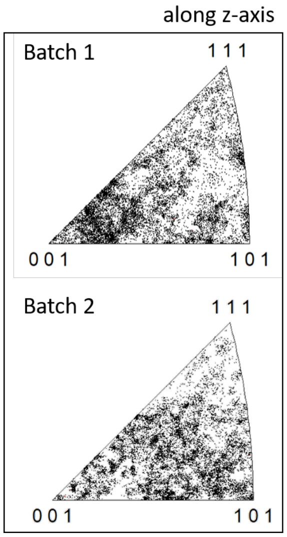

Fig. S2 IPF showing the distribution of orientations for ITO batch 1 and 2 samples along the z-axis.

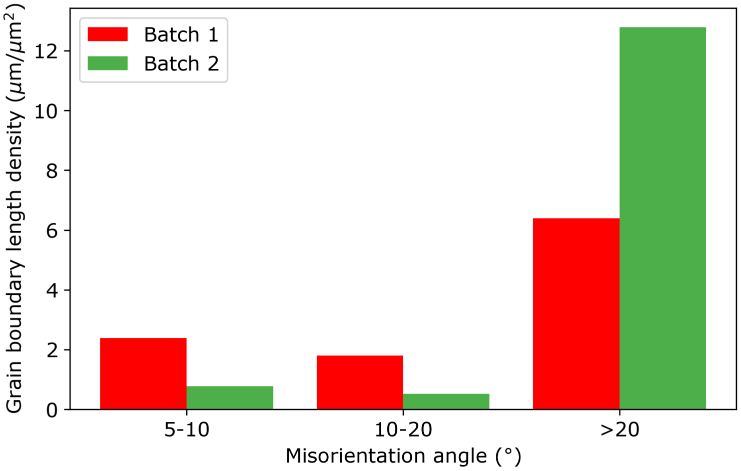

Fig. S3 Grain boundary length density for small (5-10°), medium (10-20°) and large ($>20°$) misorientation angles. The grain boundary length density is defined as the grain boundary length divided by the area of the scan.

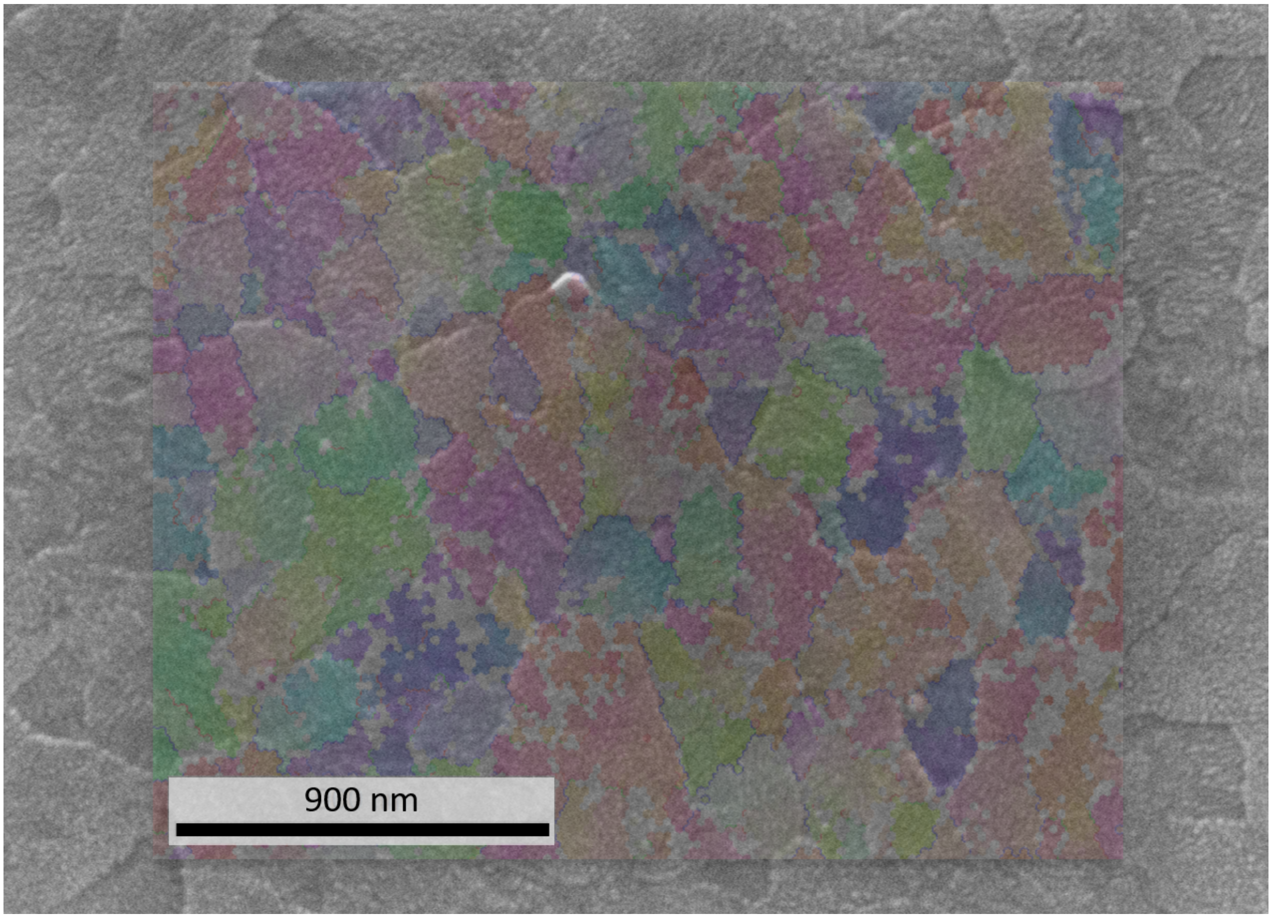

Fig. S4 Overlay of the EBSD map of ITO batch 1 as seen in figure S1a with the corresponding SEM image.

S2 Potential scheme of the double pulse technique

Figure S5 shows the potential scheme as used in the double pulse technique. First, the experiment starts with initializing for 5 seconds at OCP (of which varies between samples), followed by the nucleation and growth pulse. The lowest and highest nucleation pulse potential used are 0.2 V vs Ag/AgCl and -0.883 V vs Ag/AgCl as indicated by the green dashed and red solid line, respectively. Note that the x-axis for the nucleation pulse was stretched to highlight the pulse shape.

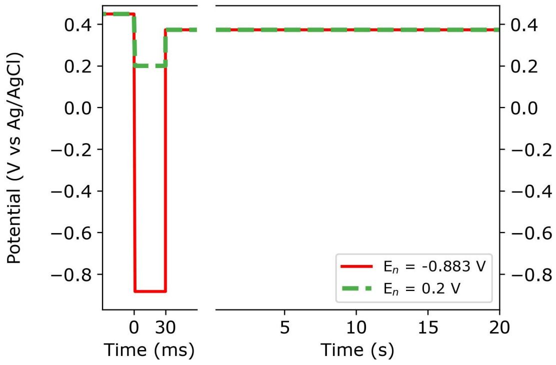

Fig. S5 Potential scheme schematic for the nucleation and growth pulse as used in the double pulse technique.

S3 Cyclic voltammograms

Figure S6 show the first and second cycle of the cyclic voltammogram of batch 1 (red) and batch 2 (green). Due to the incomplete stripping of the deposited silver, the large nucleation overpotential is significantly decreased in the second cycle of the cyclic voltammogram for both batches. This is because the silver is now also able to nucleate on the already existing silver nuclei which were formed during the first cycle. This effect is more pronounced for batch 1 than batch 2.

The experimental Nernst equilibrium potential E^0_{Ag/Ag^+} was determined by extracting the halfway potential from the second cycle of the saccharine electrolyte. Since for batch 2 most of the silver was stripped, the onset potential of the second cycle is still significant and hence the cathodic peak is shifted to more negative potentials. Therefore, for batch 2 the halfway potential of the blank electrolyte was used instead (which is very similar to the found value for batch 1). The corresponding free silver ion concentration was found to be 0.26 mM.

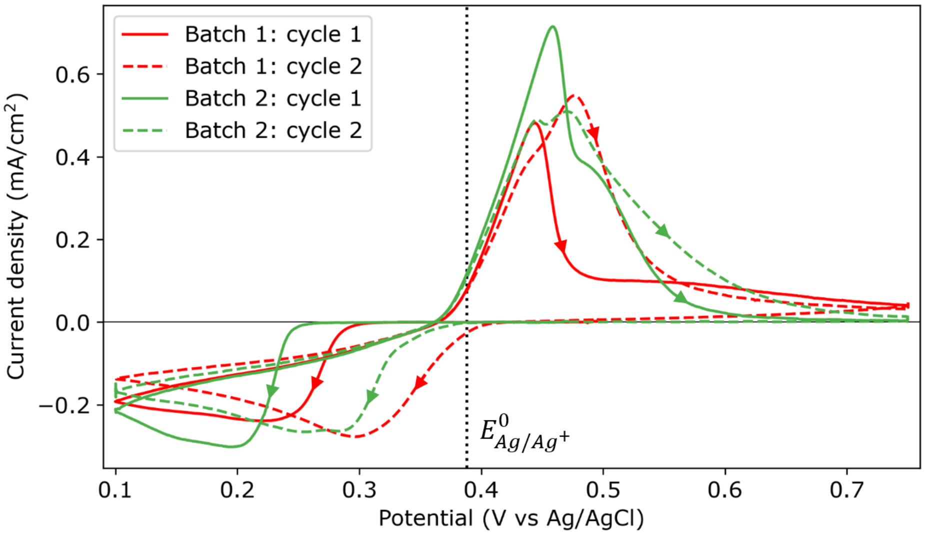

Fig. S6 Cyclic voltammogram for batch 1 (red) and batch 2 (green). The first cycles are represented by a solid line, while the second cycles are represented by a dashed line. The dotted black line indicates the experimentally found Nernst equilibrium potential.

S4 SEM data treatment

The obtained SEM images were analysed using a python script which is based on a watershed image recognition method. From these images, the particle density and size distribution were extracted. The SEM images were analysed using the OpenCV package in python (https://opencv.org/). A threshold of the raw SEM image was obtained using the *adaptiveThreshold* function. The image was then split into a sure foreground, and a sure background image. The difference between these images was used in a watershed method to determine the contour of the edges. The final contours and thus the area of the particles were obtained by using the function *connectedComponentsWithStats*. One example of an analysed SEM image is shown in figure S7. The contour of the found particles is shown in red. Particles which were lying on the outline of the image were discarded from the analyses.

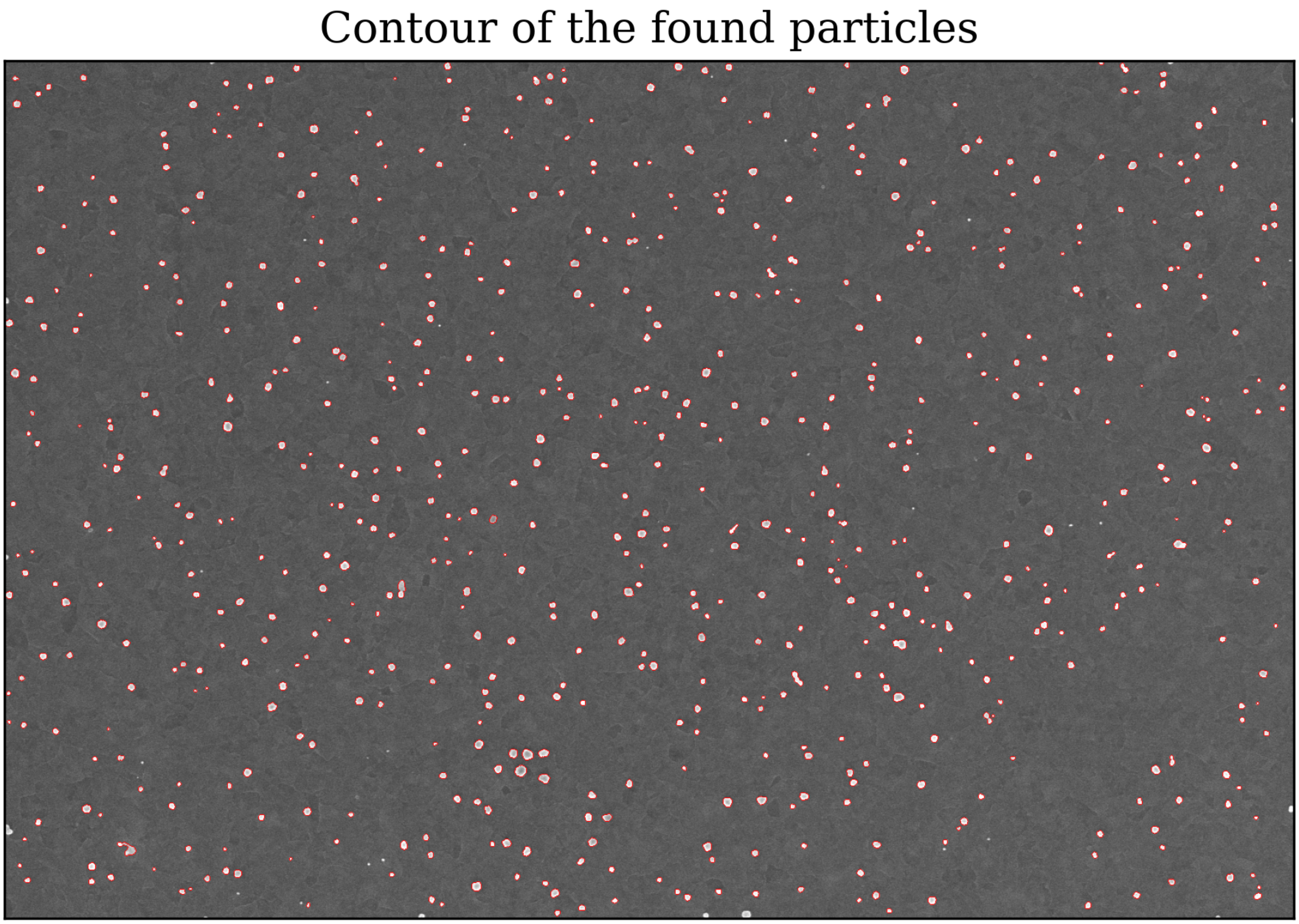

Fig. S7 Analysed SEM image from a sample corresponding to batch 1 grown at a nucleation pulse overpotential of 0.74 V. The detected particles are highlighted by the red contour.

S5 Particle size distribution

Figure S8 shows the size distribution of the selected samples shown in the SEM images in figure 3. The size distributions moves to smaller radii as the nucleation pulse overpotential increases. Although it seems that the distribution becomes more narrow, the relative standard deviation (RSD = SD/r_{mean}) is constant and might even slightly increase (especially for batch 1). For each SEM image such a distribution is obtained.

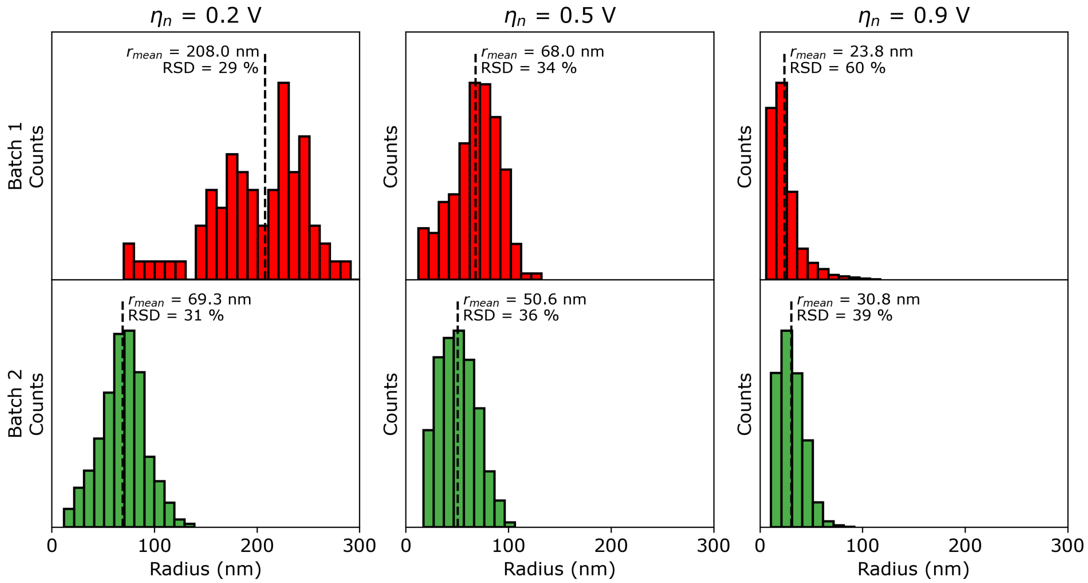

Fig. S8 Island size distribution for the selected samples as shown in figure 3. The first row shows the size distribution for batch 1 (red), and the bottom row for batch 2 (green). The mean of the distribution is indicated by the dashed black line and the value is reported next to it together with the relative standard deviation (RSD).

Per sample (applied nucleation pulse overpotential), multiple size distributions were obtained. These distributions were merged such that there is only one size distribution per nucleation pulse overpotential. From the final size distribution per nucleation pulse overpotential, one can obtain the mean island radius r_{mean}. Figure S9 shows the mean island radius as function of the corresponding island density for both batch 1 (red) and batch 2 (green). From this figure it is clear that the mean island radius is determined by the island density, irrespective of the used nucleation pulse overpotential.

The mean island radius as function of nucleation pulse overpotential is shown in figure S10. The mean island radius of batch 1 decreases with increasing nucleation pulse overpotential up to a value of 0.8 V, after which it stays constant. A similar trend for batch 2 is observed, although it is not clear whether batch 2 also reaches a constant value due to the lack of data points.

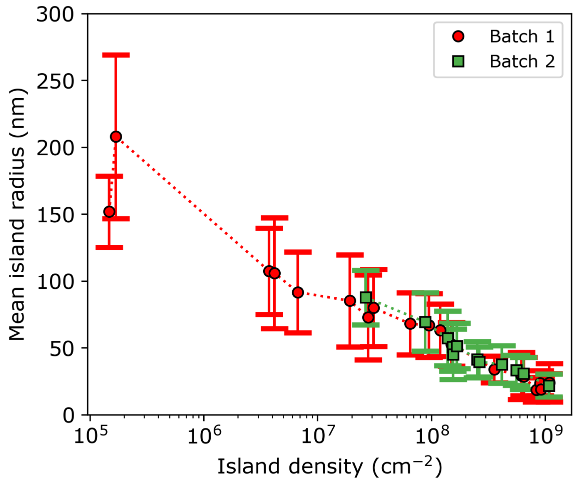

Fig. S9 Mean island radius as function of island density for batch 1 (red) and batch 2 (green). The error bars are the standard deviation of the mean values.

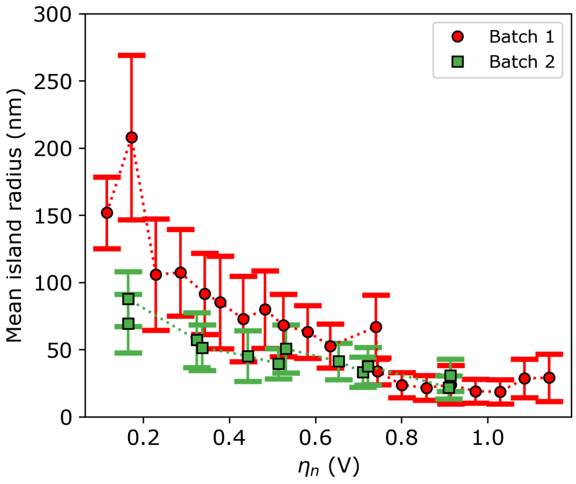

Fig. S10 Mean island radius as function of the nucleation pulse overpotential for batch 1 (red) and batch 2 (green). The error bars are the standard deviation of the mean values.

S6 Transferred charge calculation

The total transferred charge can be calculated in two ways. The first and simplest method is by integrating the current to obtain the total amount of transferred charge Q:

$$Q = \int i(t)dt \tag{S1}$$

The second method is by combining the obtained island density with the mean radius of the particles (see figures S9). From these parameters, one can calculate an effective volume by assuming that the islands are spherical.

$$V_{est} = \frac{4}{3}\pi\langle r\rangle^3 N = \frac{4}{3}\pi\langle r\rangle^3 \rho A \ , \tag{S2}$$

where $\langle r\rangle$ is the mean radius of the islands, and N is the total amount of islands which can be obtained by multiplying the island density ρ with the total exposed area A (0.95 cm^2). The total amount of deposited volume can than be converted into a expected transferred charge by using the constant of Faraday F, the molar mass M_{Ag} and density ρ_{Ag} of silver. The total expected transferred charge can then be calculated by:

$$Q_{exp} = \frac{V_{est} F \rho_{Ag}}{M_{Ag}}. \tag{S3}$$

Figure S11a shows the amount of transferred charge (deposited silver) and figure S11b shows the amount of expected transferred charge both as function of the island density, where samples from batch 1/2 are shown as circles/squares. Here, the shade of red/green of each data point indicates the nucleation pulse overpotential that was used for that sample.

In batch 1 samples, which exhibit a large spread in the island density for this nucleation potential range, two growth regimes can be discerned. At low island densities (i.e. below 10^8 cm^{-2}), the amount of transferred charge increases with island density. This behaviour agrees well with an uncoupled growth regime, where each silver islands grows independently with an unrestricted supply of Ag^+ ions. By contrast, once the island density increases beyond 10^8 cm^{-2}, ion diffusion becomes restricted by neighbouring islands, which leads to the diffusion-coupled growth regime. Given the consistently larger density of silver islands in batch 2 samples, the silver island growth is always in the diffusion-coupled regime in agreement to the behaviour observed in batch 1 samples with similar island density.

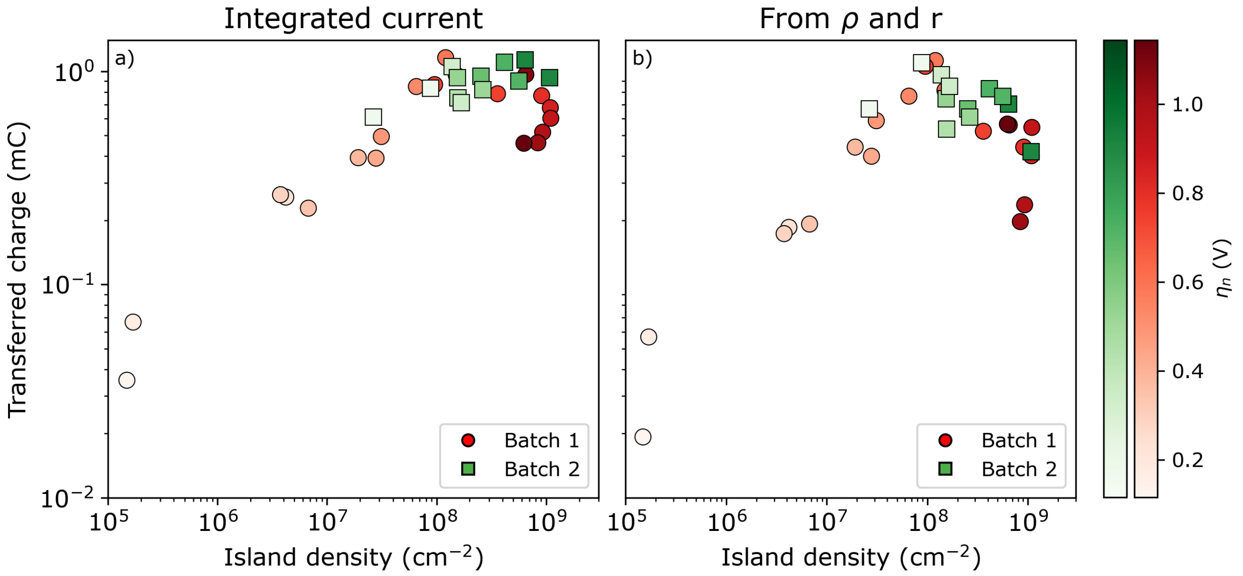

Fig. S11 a) Total transferred charge, obtained from integrating the current, as function of island density, and b) the total equivalent transferred charge, obtained from the island density and mean radius, as function of island density. The color bar indicates the used nucleation pulse overpotential for batch 1 (red) and batch 2 (green) samples.

S7 Nearest Neighbour distance analyses

In the nearest neighbour distance (nnd) analyses, the distances between each island and its nearest neighbour is compared to the expected values for a spatially random point pattern, to determine if the sample obeys complete spatial randomness (CSR). For a random point pattern, the nnd distribution should be following a Poisson distribution.

The SEM images were treated as described in section S4, from which the centroid of each island was extracted for all individual SEM images. The nnd was calculated by finding the minimum distance between the centroid of a given island and the centroid of all the other island present in that image. In order to get a decent amount of points for the histogram, the nnd of multiple SEM images per sample were combined to a single nnd histogram. The bin size of the histograms is not relevant for the calculation of the Z-score (see eq 6), since only the mean value of the nnd distribution is used. Hence, the number of bins was chosen to be 14 for the histograms shown in figure 5a) to c).

The probability density function (pdf) for a random pattern (Poisson distribution) is derived in the work of Guo *et al.*,[36] and is summarized below:

The probability of finding n particles in an area $A = \pi r^2$ is given by:

$$f(n) = \frac{(\rho A)^n}{n!} \exp(-\rho A) = \frac{(\rho \pi r^2)^n}{n!} \exp(-\rho \pi r^2) \tag{S4}$$

where ρ is the island density (assumed to be zero-volume particles).

To derive the pdf for the first order, one needs to consider the probability of finding no particles within an area of radius r from a arbitrary particle. This probability of finding zero particles within a radius r is given by (n=0):

$$f(0) = \exp(-\rho \pi r^2) \tag{S5}$$

Therefore, the probability of finding at least one particle between a radius r and $r + dr$ is then given by:

$$P(r) = 1 - f(0) = 1 - \exp(-\rho \pi r^2) \tag{S6}$$

At last the pdf $p(r)$ can be found by differentiating the equation above, and is given by:

$$dP(r) = p(r)dr = 2\pi \rho r \ \exp(-\rho \pi r^2) dr \tag{S7}$$

To to derive the pdf for the second and higher orders, one needs now to consider the probability of finding at least two particles inside a radius r, which is given by:

$$P(r) = 1 - f(0) - f(1) = 1 - \exp(-\rho \pi r^2) - \rho \pi r^2 \ \exp(-\rho \pi r^2) \tag{S8}$$

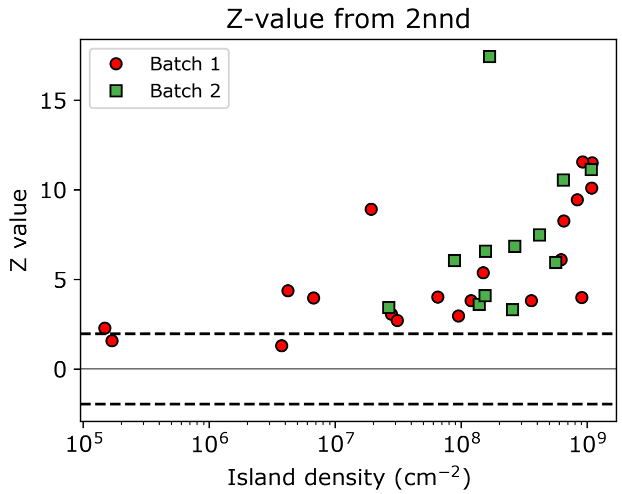

Fig. S12 Z-value obtained from individual second nnd distributions as function of the island density. Samples from batch 1 and 2 are indicated by red circles and green squares, respectively. The black horizontal dashed lines indicates the region for which the distribution of islands exhibit CSR.

and hence,

$$
\begin{aligned}
dP(r) &= p(r)dr \\
&= 2\pi\rho r\ \exp(-\rho\pi r^2)dr + 2\rho^2\pi^2 r^3\ \exp(-\rho\pi r^2)dr - 2\pi\rho r\ \exp(-\rho\pi r^2)dr \\
&= 2\rho^2\pi^2 r^3 \exp(-\rho\pi r^2)dr
\end{aligned}
\tag{S9}
$$

Once the pdf is known, the mean nnd and variance can be obtained by:

$$
\begin{aligned}
\langle r_{CSR} \rangle &= \int_0^\infty p(r) r dr \\
\sigma^2_{CSR} &= \langle r^2_{CSR} \rangle - \langle r_{CSR} \rangle^2
\end{aligned}
\tag{S10}
$$

The Z-value is defined in the main manuscript. The Z-values as function of island density for the first nnd are shown in the main manuscript in figure 6. The Z-values as function of the island density for the second nnd is shown in figure S12. The trend of both graphs are similar, indicating that sample with a higher particle density do in general not obey CSR.
